# High variability in flood discharge and stage accelerates river mobility

**DOI:** 10.1126/sciadv.adv7637

**Published:** 2025-11-28

**Authors:** Chenliang Wu, Wonsuck Kim, Shuo Yang, Frank T.-C. Tsai, Jeffrey A. Nittrouer, Tian Y. Dong, Duhwan Keum, Kyle M. Straub

**Affiliations:** ^1^ Department of Earth and Environmental Sciences, Tulane University, New Orleans, LA, USA.; ^2^ Department of Earth System Sciences, Yonsei University, Seoul, Republic of (South) Korea.; ^3^ Department of Civil and Environmental Engineering, Louisiana State University, Baton Rouge, LA, USA.; ^4^ INTERA Incorporated, Austin, TX, USA.; ^5^ Texas Tech University, Lubbock, TX, USA.; ^6^ University of Texas Rio Grande Valley, Edinburg, TX, USA.; ^7^ Earth Surface Process Modelling, GFZ German Research Center for Geosciences, Potsdam, Germany.

## Abstract

Lateral channel migration is a fundamental process in natural alluvial rivers; however, the factors that control the rate of migration remain unclear. Despite its importance in shaping river morphology, the impact of water discharge on river mobility is still largely unexplored. Here, we leverage a dataset of 64 rivers across the globe to show that higher variability in river discharge and stage promotes higher rates of river migration. To reveal the physical processes behind this relationship, we focused analyses on the lowermost 500 kilometers of the Mississippi River, where a pronounced gradient in water stage variability and migration rate exists. We demonstrate that stage variability affects channel mobility by influencing the sediment size of riverbanks and thereby controlling riverbank erodibility. These results can be used to predict river responses to climate change and decipher past hydroclimates using stratigraphy from Earth and Mars.

## Teaser

Variations in water level during overbank flooding drive lateral movement of river channels and floodplain evolution.

## INTRODUCTION

**Fig. 1.**
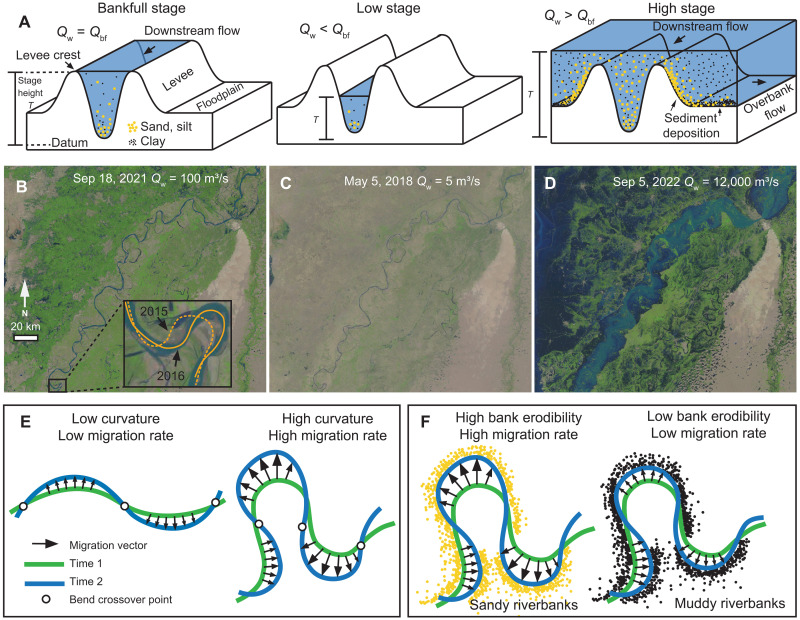
River discharge, stage, and mobility. (**A**) Conceptual diagram illustrating variability in river discharge and water stage. Bankfull discharge *Q*_bf_ is the discharge at which the water surface reaches the levee crest elevation. Bankfull stage (*T*) is the water surface elevation measured from a datum at bankfull discharge. Sediment concentration, a measure of the amount of sediment in a given volume of water, will increase as water stage increases. The levee and floodplain are inundated during flood (high stage) and receive sediments from the channel. Noncohesive sediments (e.g., sand and silt) typically settle near the levee, whereas cohesive sediment (clay) can be transported farther towards the floodplain. (**B** to **D**) satellite images of the Indus River during bankfull, low and high stages. The insert in (B) shows river migration delineated by the 2015 and 2016 channel centerlines. Channel and flood extents are typically shown in blue. Average daily water discharge can differ by orders of magnitude. (**E**) Conceptual diagram showing the influence of curvature on the channel migration rate. Arrows are schematic migration vectors indicating the local direction and magnitude of channel migration. (**F**) Conceptual illustration of the effect of riverbank material properties on channel migration: Noncohesive (sandy) riverbanks are generally more erodible than cohesive (muddy) riverbanks, producing higher migration rates.

Meandering rivers and associated floodplain environments provide important ecological habitats and agricultural and municipal land to billions of people around the world (*1*). Water discharge, a fundamental forcing that shapes river morphology (*2*–*5*), is subject to changes arising from both anthropogenic and environmental impacts. Water stage is a measure of river-water elevation referenced to a datum and typically adjusts with water discharge (Fig. 1A) (*6*). Both discharge and stage are affected by ongoing climate change that is driving extreme precipitation events, as well as droughts (Fig. 1, B to D). Collectively, these conditions result in amplified variability in river discharge and stage (*7*). Despite previous studies examining the controls of water discharge on channel morphology, the question of how increased variability in flood discharge and stage affects channel mobility, in terms of both channel lateral migration rate and riverbank erodibility (Fig. 1, E and F), remains unconstrained. This presents a challenge for infrastructure design (*8*), river restoration efforts (*9*), and land management practices (*10*).

Environmental changes that alter hydrologic regimes (*11*–*13*) and anthropogenic factors [e.g., damming (*14*)] affect stage and discharge conditions, and these influences have been linked to river mobility. Research using physical experiments have shown that higher discharge variability raises channel migration rates (*15*, *16*). In addition, during periods of the geological record that include climate perturbations (e.g., Paleocene-Eocene thermal maximum), fluvial stratigraphy indicates widespread enhancement of channel movement, consistent with higher frequency and magnitude of river flood events (*17*). More recently, a study has shown demonstrable correlation between discharge variability and floodplain reworking timescales on a global scale (*18*). The floodplain reworking timescale describes the minimum time required for the channel to rework a floodplain area that is equivalent to the channel area. This metric scales with the lateral migration rate and thus can be used to describe channel mobility (*18*, *19*). However, a physical explanation of the correlation is still lacking. Moreover, the metrics used to characterize discharge variability are not linked to the physical processes associated with overbank flooding (*20*, *21*). This limits our ability to: (i) link discharge and stage variability and fluvial depositional patterns, (ii) predict river behaviors under future environmental conditions, and (iii) reconstruct a hydroclimate record from fluvial stratigraphy.

**Fig. 2.**
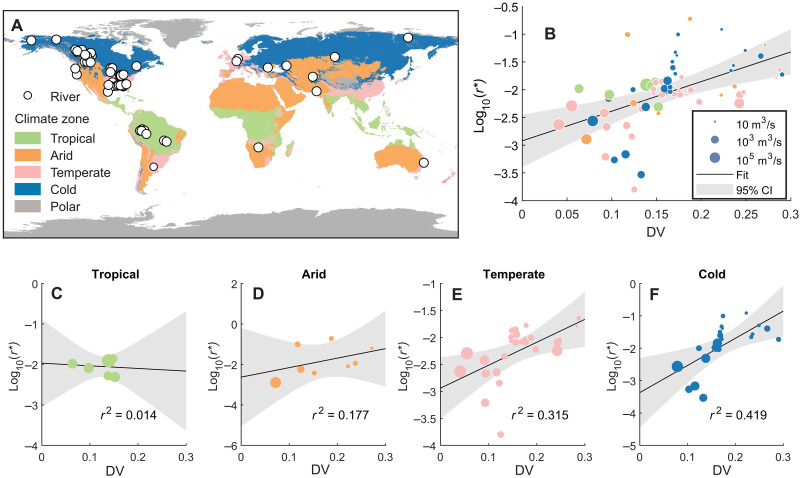
Relation between flood discharge variability and river migration rate. (**A**) Map of river discharge and migration rate data locations across climate zones. (**B**) Relation between discharge variability DV and log-transformed and width-normalized channel migration rate ( ${\text{log}}_{10}r^{*}=5.339\;\left(\pm 2.176\right)\text{DV}-2.922\;\left(\pm 0.375\right),r^{2}=0.280,p<10^{-5}$). Circle color indicates the climate zone in (A), and circle size scales with the river’s mean daily discharge. CI, confidence interval. (**C** to **F**) Relationship between DV and migration rate for rivers in each climate zone.

In this study, we first introduce a metric of flood discharge variability that focuses on overbank flood discharge and then show the linkage between flood discharge variability and river migration rate using a dataset of globally distributed meandering rivers (Fig. 2A). Second, we provide a physical explanation of how flood stage variability and river channel mobility are coupled, by focusing on spatial trends in the sediment sizes that comprise the margins of channels, and the corresponding erodibility of riverbanks using a unique and extensive dataset of riverbank material spanning the lowermost 500 km of the Mississippi River. We focus on the variability of the daily overbank flood discharge and stage rather than the whole range of these metrics because overbank flow affects sediment dispersal over the floodplain environment and therefore patterns of sediment accumulation that build natural levees and floodplain strata (Fig. 1A) (*22*, *23*). Moreover, the results of this study lend support to the hypothesis that riverbank material grain size sets riverbank strength (*24*–*26*): Fine-grain (clay) deposits form cohesive riverbanks (*27*–*30*), strengthening them and decreasing erodibility (*25*), which in turn reduces channel mobility (*31*, *32*).

## RESULTS

### Flood discharge variability and river migration rate

To demonstrate the impact of overbank floods on river mobility, we quantify the flood discharge variability DV of a river as

$$\text{DV}=\frac{1}{n}\sum_{i=1}^{n}\sigma_{Q\left(i\right)}/\mu_{Q\left(i\right)}$$ (1)

where *n* is the number of years of discharge record, σ*_Q(i)_* and μ*_Q(i)_* are the SD and mean of the daily flood discharge ${Q_{w}|}_{Q_{w}>Q_{\text{bf}}}$ (m^3^/s) for a given year *i*. Bankfull discharge $Q_{\text{bf}}$ is approximated as the 1.5-year returning flood discharge (*2*, *4*, *5*). Years without discharge greater than bankfull discharge are ignored for the calculation as no sediments were transported to floodplain. Therefore, DV is essentially the mean annual coefficient of variation of overbank flood discharge, which is a statistical measure of the SD as a percentage of its mean.

River lateral migration rates are compiled from previous work (*33*, *34*). The migration rate is calculated on the basis of the movement of a river centerline and normalized by the width of the channel *B* (meters) (*34*). River centerlines are first generated from historical maps, aerial photos, and satellite images. Lateral migration rates *r* (meters per year) can then be calculated on the basis of centerline locations at two different times using two methods: (1) dynamic time warping (DTW) (*35*) or (2) the ratio between the area swept by a channel and the channel length, divided by the time duration (*33*). The DTW method correlates discretized points on river centerlines from different times so that local migration rates can be calculated (Fig. 1E). It provides comparable estimates as method 2 when local migration rates are averaged over bend scales. Both methods are applied over different time periods and river reaches to provide reach- and time-averaged lateral migration rates r* (*r/B*). We also compiled a dataset of daily discharge records for 64 globally distributed rivers of various sizes where the mean discharge of the rivers spans four orders of magnitude (Fig. 2B). The gauging stations selected are in proximity to where the lateral migration rate measurements were made (*33*, *34*).

This dataset reveals a positive relation between DV and width-normalized river migration r* (Fig. 2B). The relation is not obvious for rivers in tropical zones, likely due to the low number of samples (*N* = 7) and the limited ranges of DV and r* for the tropical rivers documented (Fig. 2C). However, this positive relation is pronounced for rivers within other climatic zones (*36*), including arid (*N* = 8), temperate (*N* = 26), and cold regions (*N* = 22) (Fig. 2, D to F). This statistically significant correlation provides evidence to support the hypothesis that discharge variability is a key control on the river migration rate (*15*, *33*). Although there is substantial variability in this relationship, DV presents a controlling factor that explains almost 30% of the total variance in r*, which is higher than or at least comparable to other factors, including sediment supply (*34*). Similar hypotheses have been proposed by previous research (*16*–*18*), but we still lack a physics-based explanation. Since the metric DV describes the variability of overbank floods, which are responsible for transporting sediment to build riverbanks and floodplains, we further hypothesize that variability of discharge and stage could affect river mobility by setting the composition and thus the erodibility of riverbanks.

### Flood stage variability and migration rate of the lowermost Mississippi River

**Fig. 3.**
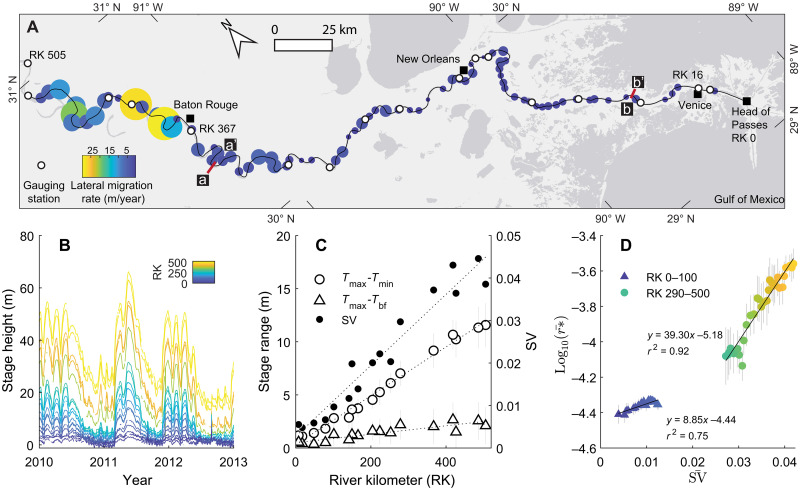
Planform morphology, water stage history, and migration rate of the LMR. (**A**) Map of the LMR showing channel path (RK 0 to 505) and bend-averaged migration rate. Migration rate is color-coded, and circle size scales with the rate. (**B**) Stage record from 17 gauging stations. Stage records are color-coded by RK of each station. The length of stage records ranges 17 to 70 years, while the plot shows data from 2010 to 2013 for illustrative purposes. (**C**) Spatial trends in stage range and stage variability SV. (**D**) Correlation between reach-averaged stage variability SV and width-normalized migration rate. Data points are color-coded by RK.

We test this hypothesis using a single river—the lowermost Mississippi River (LMR). The LMR is the section that extends from Head of Passes at the outlet located at river kilometer (RK) 0 to approximately RK 500. Previous work showed that the channel lateral migration rate of the LMR increases from the outlet moving upstream (Fig. 3A) (*37*, *38*). This reach of the Mississippi River also naturally develops a condition of spatially varying flow stage, which is a common hydraulic condition for the terminal reach of lowland rivers called the “backwater effect” (*37*, *39*, *40*). Stage varies more in the upstream compared to the downstream section as stage equilibrates to the sea level toward the outlet (Fig. 3, B and C). The backwater condition also causes a systematic gradient in channel hydraulics (e.g., flow velocity and flow depth) during low stages (*37*, *41*). However, the channel develops normal flow conditions with quasi-uniform reach-scale hydraulics during flood stages when the channel is considered morphodynamically active. For example, levee development and riverbank retreat typically take place during overbank flooding (*42*, *43*). This condition makes the LMR an ideal place to test the control of stage variability on river mobility, as other parameters that might influence river mobility during flood [e.g., flow velocity, sediment supply (*34*, *44*), vegetation type, density (*45*, *46*), etc.] of the LMR are approximately uniform. The only major exception is the presence of partially exposed and compacted Pleistocene and late Holocene mud in the channel sidewalls in the lower 300 km of the LMR (*47*, *48*) (Supplementary Materials). The substratum is more erosion resistant compared to riverbank materials deposited from recent alluvial processes, so that the presence of substratum can lower the lateral migration rate. Therefore, we will distinguish the impact of sidewall substratum from the control of stage variability on channel mobility in the following analysis.

We compiled daily stage records from 17 gauging stations along the LMR (Fig. 3, A and B). There is a systematic increase in stage variability with distance upstream (Fig. 3B). For example, the range of stages, defined as the difference between maximum and minimum annual stages (*T*_max_ and *T*_min_), increases upstream. The range of flood stages, defined as the difference between the maximum annual stage and bankfull stage (*T*_bf_), also increases upstream (Fig. 3C). This suggests increased variability in water stage as the SDs of the stage ranges (*T*_max_ − *T*_min_) and flood stages (*T*_max_ − *T*_bf_) also increase upstream (Fig. 3C). To quantitatively evaluate the stage variability of the LMR, we introduce a metric, stage variability (SV)

$$\text{SV}=\frac{1}{n}\sum_{i=1}^{n}\sigma_{T\left(i\right)}/\bar{H_{\text{bf}}}$$ (2)

where *n* is the number of years of stage record, σ*_T(i)_* is the SD of stage for the overbank flow ${T|}_{T>T_{\text{bf}}}$ for a given year *i*, and $\bar{H_{\text{bf}}}$ is the reach-averaged bankfull flow depth. The structure of the metric SV is similar to that of DV, as both quantify the variability of their associated physical quantities. SV is similar to the coefficient of variation but slightly different, as it measures the SD of overbank flood stage as a percentage of reach-averaged bankfull flow depth at each gauging station instead of the average of stage. This parametrization approach accounts for the fact that the flow depth is absolute, regardless of the choice of datum for stage measurements. Since stage measurements are relative to a datum and can even be negative, average stage values are not suitable for calculating the coefficient of variation, especially when comparing stage records from different stations.

We calculated stage variability for all 17 gauging stations. The calculated SV values were evaluated against RKs and fit with a linear regression function to estimate the moving average of stage variability $\bar{\text{SV}}$ for each channel bend (Fig. 3C). There is a positive relation between $\bar{\text{SV}}$ and the moving averaged annual migration rate $\bar{r^{*}}$ (Fig. 3D), supporting the hypothesis that high stage variability enhances river mobility in terms of lateral migration rate. This relation is pronounced for both upstream (RK 290 to 500) and downstream sections (RK 0 to 100). The difference in regression trends between the two sections is attributed to the presence of sidewall substratum in the downstream section, where substratum consistently comprises 9% of the channel sidewall (*47*). The spatially consistent proportion of substratum in the channel sidewalls suggests that the gradient of stage variability is the dominant control on spatial variations in the channel migration rate. The river section from RK 100 to 290 is excluded from this analysis because the percentage of sidewall substratum in this reach is spatially variable, making it difficult to isolate the impact of stage variability on river mobility.

### Riverbank material, depositional pattern, and erodibility

**Fig. 4.**
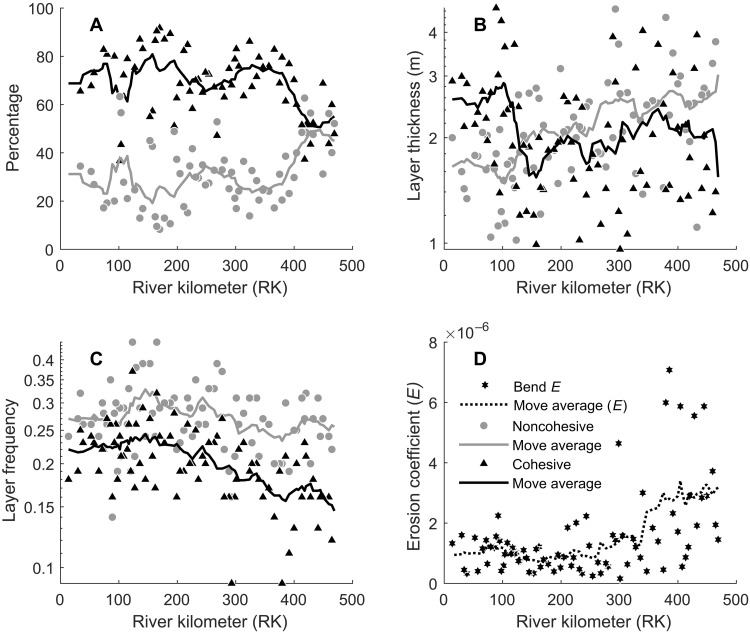
Systematic trends in riverbank materials and depositional patterns. (**A**) Bend-averaged percentages of cohesive and noncohesive riverbank materials along the LMR. (**B**) Bend-averaged riverbank sediment layer thickness. (**C**) Bend-averaged riverbank sediment layer frequency. (**D**) Calibrated bend-averaged riverbank erosion coefficient. Moving average values are calculated over 10 channel bends.

To provide a mechanism behind the control of stage variability on channel mobility (i.e., channel migration rate and riverbank erodibility), we document grain-size distributions for the riverbank materials along the LMR using 1656 geotechnical borings and calculate riverbank erodibility of the LMR using a meandering river model that accounts for the influence of the spatially variable channel curvature on the migration rate (fig. S1; see the Supplementary Materials for details). Spatial changes in riverbank material size and sediment accumulation patterns are measured for both outerbanks and innerbanks (fig. S3), whereby the latter is typically associated with point bars. We only focus on outerbanks (i.e., cut banks) in this study (Fig. 4). In this study, sand and silt are categorized as noncohesive material, and clay is categorized as cohesive material, based on their mechanical property (Supplementary Materials). Cohesive material percentage averaged over a channel bend ranges from 92% (at RK 170) to 37% (at RK 418) for the studied river reach (Fig. 4A and fig. S3). A moving average of the cohesive material percentage shows an overall upstream decrease particularly in the upper section (RK 327 to RK440) from 79 to 51%.

The riverbank consists of alternating layers of both cohesive and noncohesive sediments (Fig. 2, D and E). The moving average layer thickness for noncohesive sediments increases by 100% upstream, from 1.5 to 3 m (Fig. 4B). Therefore, noncohesive sediment layers thicken moving upstream. The moving average layer thickness for cohesive sediment layers is more spatially variable: It decreases upstream from 2.8 m (RK 103) to 1.5 m (RK 145) and then increases upstream to 2.4 m at RK 366.

Layer frequency represents the number of sediment layers per unit riverbank height. It is calculated as the ratio between the number of sediment layers in a riverbank and the riverbank thickness, from the depth of the local thalweg to the adjacent levee crest. The moving average layer frequency for cohesive material shows an overall 40% upstream decrease from 0.243 (RK145) to 0.146 (RK 469) (Fig. 4C). The moving average layer frequency for noncohesive sediments also shows an overall upstream decrease from 0.328 (RK145) to 0.233 (RK 379). There are more sediment layers in the riverbank per unit riverbank height in the downstream reach compared to the upstream reach, especially for cohesive material.

The riverbank erosion coefficient, *E*, is a scaling term that measures how easily the riverbank is eroded (see Materials and Methods for details), so that the higher the *E* value, the more erodible the riverbank. This coefficient shows a factor of 40 differences (0.16 × 10^−6^ to 7.1 × 10^−6^) between individual channel bend values. The upstream section of the studied river reach (RK 250 to RK 500) (Fig. 4D) shows a threefold increase in the moving average riverbank erosion coefficient (from 0.84 × 10^−6^ to 3.18 × 10^−6^). The downstream section RK 0 to RK 250 shows relatively low riverbank erosion coefficients, ranging from 0.25 × 10^−6^ to 2.25 × 10^−6^.

### Stage variability regulates sediment supply to levee

**Fig. 5.**
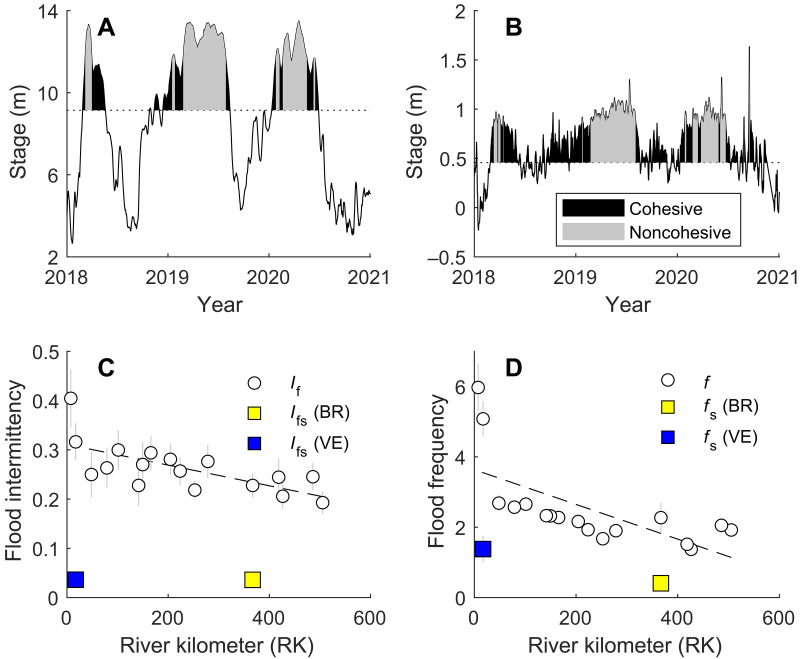
Sediment supply through overbank flood flow. Stage history at Baton Rouge (BR) (**A**) and Venice (VE) (**B**) (locations shown in Fig. 3A). Dashed line marks the bankfull stage before the construction of artificial levees. Gray shading marks river stages with notable suspended sand in the overbank flow column. Black shading marks stages with only clay in suspension in the overbank flow column. (**C**) Spatial trends in flood intermittency *I*_f_ and intermittency for sand-transporting overbank floods *I*_fs_. (**D**) Spatial trends in flood frequency *f*_s_ and frequency of sand-transporting overbank floods *f*_s_. Error bars represent the SD of the bootstrapped mean.

To elucidate the relationship between stage variability and riverbank properties (i.e., material size, depositional pattern, and erodibility), we calculate flood intermittency *I*_f_ and frequency *f* and estimate the sediment sizes supplied to levees using the Rouse model (*49*). Flood intermittency measures the fraction of time that flow stage is higher than the natural bankfull stage (*50*), and flood frequency measures the average annual count of overbank flood events. In our analysis, a flood event is defined by a stage peak that exceeds the natural bankfull stage on the time series of daily stage record (Figs. 3B and 5, A and B). These metrics set limits on the duration and frequency of overbank flow that transports sediments, particularly clay, to build riverbanks. This is because clay tends to be uniformly suspended throughout the flow column and is therefore supplied to the levees during all overbank flow conditions (*51*, *52*).

**Fig. 6.**
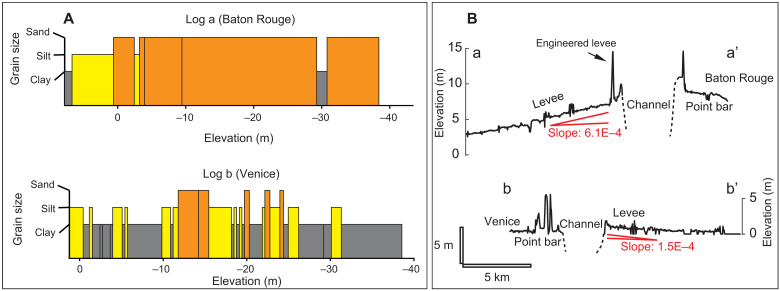
Riverbank depositional pattern and levee geometry of the LMR. (**A**) Representative stratigraphic logs (a) and (b) from riverbanks near BR and VE, respectively. The BR log shows thicker sedimentary layers, whereas the VE log shows thinner sedimentary layers. See fig. S1 for locations of the logs. (**B**) Levee profiles near BR and VE. Locations of the profiles are marked by red lines in Fig. 3A. Spikes in the profiles represent engineered levees and are excluded when calculating the transverse levee slope.

Stage records from 17 gauging stations along the LMR are used to calculate flood stage, assuming that the records are representative for the period when the river established its current course around 2800 years ago (*53*). Because of the construction of engineered levees, the river stage above natural bankfull stage was artificially raised (*50*); therefore, our calculation of flood characteristics presents the upper limit of the estimate. The natural bankfull stage is determined by considering the geometry of the rating curve (daily discharge versus stage), which yields comparable results as a previous study (fig. S2) (*54*). Stage records reveal that overbank floods shorten in duration and become less frequent with distance upstream as flood intermittency and flood frequency decreases (Fig. 5, C and D). Therefore, the spatial trends in flood intermittency and flood frequency suggest that less clay was supplied to the levees, and there was less frequent deposition of clay layers with upstream distance (Figs. 4, A to C, and 6).

To estimate sand supply to levees, we performed Rouse modeling at an upstream [Baton Rouge (BR), RK 388] and a downstream [Venice (VE), RK 16] gauging station, treating them as end-member cases representing high and low stage variability scenarios, respectively (see Materials and Methods for details). The BR station is the only station with a daily discharge record that is required for the Rouse model, and the discharge at the VE gauging station is assumed to be the same as BR for simplicity. Channel hydraulics induced by backwater effects, such as spatial and temporal variations in flow velocity, are compiled from a previous study (*37*) and are incorporated in the Rouse model to simulate sediment concentration profiles given different stage heights at each station.

Results from the Rouse sediment concentration model are first used to calculate intermittency (*I*_fs_) of overbank flood events that transport quantifiable suspended sand loads to the levees. Sand supply to levees is only quantified when the modeled volumetric sediment concentration (dimensionless) in the overbank flow exceeds a minimum value based on an instrumental detection limit (1.89 × 10^−6^). Notably, this intermittency value (*I*_fs_) is the same at both BR (0.04) and VE (0.04) (Fig. 5C) (see Materials and Methods for details). However, the VE stage record shows a flood intermittency *I*_f_ (0.31) of about 34% higher than BR (0.23). While clay can be transported to levees whenever overbank flow occurs, sand is only transported above certain stages (Fig. 5, A and B). Therefore, the percentage of sand supply compared to total sediment supply should be higher at BR. This likely resulted in the development of much sandier riverbanks in BR than VE (Figs. 4A and 6A). This is also supported by the modeled average suspended sand concentration of the overbank flow, which is 70% higher for BR (1.08 × 10^−5^) compared to VE (6.32 × 10^−6^). The difference in sand supply to levees likely also resulted in the difference in transverse levee geometry at BR and VE (Fig. 6B): The sandier BR levee is four times steeper than the muddier VE levee.

The flood stage frequency *f* is 2.27 at BR and 5.08 at VE (Fig. 5D). Flood events, which could supply sand to the preartificially leveed overbank, occur at a lower frequency (*f*_s_) at BR (0.41) compared to VE (1.38) (Fig. 5D). Therefore, depositional events for sediment layers of both sand and clay will reduce with distance upstream between VE and BR. This agrees with the observations of more frequent sand and clay layers from the boring record with distance upstream (Figs. 4C and 6).

## DISCUSSION

The extensive riverbank material dataset taken together with the overbank sediment supply analysis demonstrates a linkage between flow-stage variability and material composition of the riverbank and its erodibility. When overbank flood events occur with high stage variability, a relatively greater proportion of sand is transported from the channel to the riverbank (Fig. 5A). This facilitates deposition of thick layers of noncohesive materials on the riverbanks (Figs. 4, A to C, and 6A). The upstream reach of the LMR that is susceptible to relatively high stage variability is also associated with less frequent flooding of the riverbank (*40*). In contrast, the water level shows low variability and fluctuates around bankfull stage for the downstream reach of the LMR, resulting in more frequent floods (Fig. 5B) and relatively finer sediment layers in the riverbank (Fig. 4C). These assessments suggest that stage variability can affect both the supply and frequency of cohesive and noncohesive materials delivered to the riverbank. The supply of sediment, in particular its size, also determines the depositional pattern and transverse geometry of the levees (Fig. 6B) (*23*). The key characteristics of riverbanks, especially cohesiveness and heterogeneity, present important controls on riverbank erodibility and associated channel mobility.

**Fig. 7.**
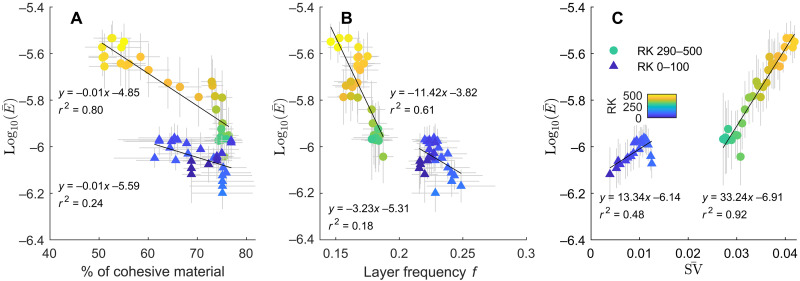
Controls of materials, depositional patterns, and stage variability on riverbank erodibility. (**A**) Correlation between moving-averaged riverbank erosion coefficient and cohesive material percentage. (**B**) Correlation between moving-averaged layer frequency and riverbank erosion coefficient. (**C**) Correlation between moving-averaged stage variability and riverbank erosion coefficient. Error bars represent the SD of the bootstrapped mean.

First, we determine negative correlations between the moving averages (over a spatial scale of 10 channel bend segments) in the percentage of cohesive material in the riverbanks and the riverbank erosion coefficient $\bar{E}$(Fig. 7A). These correlations demonstrate that the riverbank erosion coefficient increases as cohesive material building the riverbank decreases, as is found from downstream to upstream in the LMR. Complementing previous physical experiments (*31*, *32*), this study lends support to the hypothesis that, in a alluvial river system, riverbanks are strengthened and erosion is reduced (*25*–*27*) as the fraction of cohesive material comprising the riverbank increases, due to the associated higher shear stress required for sediment entrainment (*29*, *30*).

The downstream (RK 0 to 100) and the upstream (RK 293 to 469) sections follow two separate trends (Fig. 7A). This reflects the influence of the partially exposed and compacted Pleistocene and late Holocene mud in the channel sidewalls for the downstream section RK 0 to 100 (fig. S1) (*47*). The presence of sidewall substratum in the riverbank could lower the overall riverbank erosion coefficient in addition to the control of riverbank material deposited by alluvial process (i.e., natural levee). For example, at the same percentage of cohesive materials, the downstream section shows lower erosion coefficients, demonstrating that the presence of sidewall substratum is lowering the riverbank erodibility. Since the distribution of sidewall substratum in riverbanks is spatially uniform over RK 0 to 100 (*47*), the spatial variations in riverbank erosion coefficient for the downstream section can partly be attributed to the variations in riverbank materials including both compacted and unconsolidated sediments.

Second, there is a negative correlation between the moving averages (over a spatial scale of 10 channel bend segments) in the layer frequency of cohesive material and the riverbank erosion coefficient $\bar{E}$ (Fig. 7B), especially for the section that spans RK 293 to 469, where the riverbank erosion coefficient also shows the greatest change (Fig. 4D). Higher layer frequency of cohesive material suggests a heterogeneous riverbank structure. This could lower riverbank erosion due to cantilever failures, slumps, and seepage erosion, which typically develop through overhanging levee tops or oversteepened riverbank walls (*43*, *55*, *56*). Previous experiments have demonstrated that levees with more cohesive material are not as susceptible to failures (*55*). Moreover, previous studies have suggested that slump blocks could cover riverbank toes and defer riverbank erosion (*56*, *57*). Therefore, slump blocks with more cohesive or heterogeneous materials will also likely slow riverbank erosion furthermore.

Last, there is a positive correlation between the moving averages in stage variability $\bar{\text{SV}}$ and riverbank erosion coefficient $\bar{E}$ (Fig. 7C), supporting the hypothesis that variability of stage enhances river mobility by setting the composition and thus the erodibility of riverbanks. Because of the influence of sidewall substratum, the riverbank erosion coefficient is less sensitive to changes in stage variability in the downstream section (RK 0 to 100) compared to the upstream section (RK 293 to 469).

### Implications

It has been widely recognized that ongoing climate change is resulting in more extreme droughts and precipitation events (*7*, *58*, *59*), thus shifting the hydrological regime of river systems globally (*60*, *61*). These conditions will result in greater variability in river discharge and stage that, as shown in the analysis presented herein, favors transport of noncohesive material to river levees and adjacent floodplain environments. As a consequence, channel mobility will increase as the concentration of this material decreases riverbank sediment strength (*33*). To assess how changing stage conditions affect channel mobility, we estimate a timescale for riverbank material replacement. This is assumed to be set by the characteristic time for the channel to migrate laterally one channel width (lower bound) and one channel-belt width (upper bound). For the Mississippi River, the average channel width is 1000 m (*37*), and the migration rate varies spatially, averaging ~50 m/year (between RK 500 and 1700) and <~ 5 m/year (RK 0 and 500) (*38*). Therefore, estimates for the minimum (lower) timescale of riverbank material replacement range 20 to 200 years. Using a ratio of 20 for channel-belt width to channel width (*62*), the maximum (upper) estimate for riverbank material replacement is 400 to 4000 years. This exercise is applied to a global dataset of 139 rivers (*34*). It is determined that the 90th percentile for lower and upper ages ranges 6 to 192 and 120 to 3840 years, respectively. This provides an order-of-magnitude estimation for riverbank material replacement and indicates that adjustments to channel mobility for most river systems in response to changing stage variability could arise over decades to millennia. These timescales are consistent with estimates of other metrics for characterizing rates for floodplain turnover and sediment age (*63*–*65*). Although portions of the Mississippi and many other meandering rivers are typically modified by engineering practices to restrict lateral movement, many rivers, particularly in the global south, remain unencumbered. In addition, because rivers tend to preferentially erode younger deposits (*66*, *67*), we argue that the pace of sediment replacement to coarser, unconsolidated channel and riverbank material could be faster than estimated.

The results of this study are consistent with recent work that show how a change in hydrological regime results in a fundamental shift in fluvial stratigraphy, from a floodplain dominated by mudstone to an architecture where channel-belt sandstones are pervasive (*16*, *68*, *69*). This arises as the material size and layer frequency of ancient fluvial deposits represent a feedback between stage variability and riverbank development. For example, older riverbank sediments are removed when a channel moves laterally. If stage variability increases, then the overbank flow supplies more noncohesive materials to the riverbank. As a result, the percentage of cohesive material and sediment layer frequency in the riverbank reduces; as a consequence, there is an increase in riverbank erodibility and channel mobility. More sand supply to the riverbank will generate higher and steeper levee slopes as sand readily settles compared to mud (*23*). For example, the transverse levee slope at BR is about four times steeper compared to VE (Fig. 6B). This results in a positive feedback: Taller levees require higher stage to inundate; thus, the channel transports a higher percentage of sand to levees. This implicates variable stratigraphy as a possible way to assess stage variability and associated hydrological regimes for ancient systems (*17*), without necessitating adjustments in other external forcings such as sediment supply and/or tectonics (*70*, *71*). The spatial pattern of the LMR channel sinuosity and channel-belt width near coasts are not unique and are also found on Mars (*33*, *72*–*75*). These geomorphic and sedimentological features provide evidence for the presence of ancient martian ocean and fluvial deltaic systems. Moreover, these features could provide insights into the channel and riverbank dynamics of ancient martian river systems and are thus useful for linking the stratigraphic records to the past hydrological regimes (*76*).

## MATERIALS AND METHODS

### Historical channel paths and channel migration rate

Historical channel paths from 1883 and 1913 were reconstructed to calculate the channel lateral migration rate during this 30-year interval. The Mississippi River Commission Comprehensive Mississippi River Surveys of 1913 and 1883 (scale 1:20,000) were accessed from US Army Corps of Engineers site (https://www.mvn.usace.army.mil/Missions/Engineering/Geospatial-Section/MRHB_Historic/). These surveys document channel morphology before major human modifications (*50*). The maps from each survey were georeferenced in QGIS. Banklines from both surveys were traced manually. Centerlines were then found using Voronoi polygons. The centerlines were smoothed in MATLAB using the smooth function with the Savitzky-Golay method.

Individual channel bends were constrained by identifying the succeeding inflection points, where centerline curvature becomes zero, in the along-stream direction. Centerlines were discretized over 100-m intervals. We first use a DTW method (*35*) to calculate the migration rate for each discretized point along the centerline. The bend migration rate was then calculated by averaging the migration rates of all the points within the bend.

### Rouse modeling

Volumetric sediment concentration ε_s_ in the flow column at the BR and VE gauging stations is calculated using the Rouse model (*49*)

$$\frac{\varepsilon_{s}}{1-\varepsilon_{s}}={\left(\frac{\varepsilon_{s}}{1-\varepsilon_{s}}\right)}_{Z=a}{\left[\left(\frac{H-Z}{Z}\right)\left(\frac{z_{a}}{H-z_{a}}\right)\right]}^{p}$$ (3)

where *a* is the elevation of the top of the saltating bedload layer, *H* is the flow depth, *Z* is a depth of interest within the flow column where the sediment concentration is calculated, and *z_a_* is the thickness of the bedload layer, calculated by (*77*)

$$\frac{z_{a}}{D_{50}}=\frac{A_{1}\frac{\tau_{b}}{\tau_{\text{cr}}}}{1+A_{2}\frac{\tau_{b}}{\tau_{\text{cr}}}}$$ (4)

where *D*_50_ is the median grain diameter, τ_b_ is the boundary shear stress, τ_cr_ is the critical shear stress for *D*_50_, *A*_1_ = 0.68, and *A*_2_ is calculated by

$$A_{2}=0.0204\left(\text{ln}D_{50}\right)+0.0709$$ (5)

The sediment concentration at the top of the bedload layer is calculated as (*78*)

$$\varepsilon_{s\left(Z=a\right)}=\frac{0.004\lambda E_{t}}{1+0.004E_{t}}$$ (6)

where λ is the bed sediment concentration set at 0.65, *E*_t_ is the transport stage calculated as $E_{t}=\left(\frac{\tau_{b}}{\tau_{\text{cr}}}-1\right)$.

The Rouse number *p* in Eq. 1 is calculated by

$$p=\frac{\omega_{s}}{\alpha \kappa u_{\text{sf}}^{*}}$$ (7)

where ω_s_ is the settling velocity of grain size determined by the method of Dietrich (*79*), κ is the clear water von Karman constant (0.41), $u_{\text{sf}}^{*}$ is the skin friction shear velocity, and α is the density stratification adjustment, assumed to be 0.8 for simplicity (*80*).

To calculate the daily sediment concentration using Eqs. 1 to 5, skin friction shear velocity and boundary shear stress need to be specified. For BR, daily depth-averaged flow velocity (*u*) was calculated by interpolation using the stage and velocity relation from US Army Corps of Engineers (USACE) (https://rivergages.mvr.usace.army.mil/WaterControl/Districts/MVN/velo_br.htm). Skin friction shear velocity is calculated as $u_{\text{sf}}^{*}=u\sqrt{C_{f}}$ , where *C*_f_ is the friction coefficient. Empirical relation between discharge (*Q*_w_) and skin friction was constructed on the basis of published records (*37*). Then, daily skin friction for both BR and VE was calculated on the basis of the empirical relation and daily discharge record at BR. Bed shear stress is calculated as $\tau_{b}=\rho C_{f}u^{2}$ , where ρ is the water density (1000 kg/m^3^). Since there is no daily discharge record at VE, we first constructed the rating curve for VE, assuming that VE shares the same daily discharge as BR. An exponential relation was fitted for the rating curve. Then, daily discharge for VE was recalculated on the basis of the regression fit and daily stage record. Daily depth-averaged flow velocity was reconstructed using regression fit based on published data (*37*). Then, daily reach-averaged flow depth for VE is calculated as $H=\frac{Q_{w}}{\mathrm{uB}}$ , where *B* is the channel width set at 827 m (*37*). Median grain diameters at BR and VE are set at 300 and 250 μm, respectively (*37*).

Bankfull stages for BR and VE were set at 30 and 1.5 ft, corresponding to flow depths of 15.5 and 17.2 m, respectively. These represent the natural bankfull stages before engineered levee enhancement (height increase), which raises bankfull stages (*50*, *54*). Then, average sediment concentration in the overbank flow (flow above bankfull stage) is calculated. If the average sediment concentration is greater than the instrumental detection limit for sand (ε_s_ = 1.89 × 10^−6^) (*22*), then the flow is considered to be sand-transporting flood. The average sediment concentration in the overbank flow sets the upper limit for sediment supply to the levee (*23*).

### Bank erosion coefficient

In this study, we use the HIPS model (*56*) to reconstruct riverbank erodibility. In the HIPS model, dimensionless riverbank erosion coefficient *E* is used to characterize riverbank erodibility (*81*, *82*). Lateral migration is considered as a result of cut riverbank erosion and calculated as

$$\zeta =Eu_{b}$$ (8)

where ζ is the lateral migration rate, *h* is the reach-averaged flow depth, and *u*_b_ is the excess flow velocity near riverbank. The excess flow velocity *u*_b_ can be calculated by

$$u_{0}\frac{\partial u_{b}}{\partial s}+2\frac{u_{0}}{h}C_{f}u_{b}=b\left[-u_{0}^{2}\frac{\partial \xi }{\partial s}+C_{f}\xi \left(\frac{u_{0}^{4}}{gh^{2}}+A\frac{u_{0}^{2}}{h}\right)\right]$$ (9)

where *u*_0_ is the reach-averaged flow velocity, *s* is the streamwise distance, *b* is the reach-averaged half bankfull flow width, *C*_f_ is the friction coefficient, ξ is the local curvature of channel centerline, and *A* is a constant slope factor. We followed the HIPS model implementation method (*83*) to calculate *u*_b_ at points discretized from the Mississippi River centerline. The lateral migration rate at each point can then be calculated using Eq. 1. Reach-averaged flow depth and channel width at each point are calculated on the basis of local depth and width measurements. Bend migration rate ζ_c_ is calculated as the average of lateral migration rate of all the points within the bend. A grid search method for *E* was performed over the range of 1 × 10^−7^ to 1 × 10^−6^, and corresponding bend migration rates were calculated accordingly. Measured bend migration rate ζ_m_ from historical maps and calculated bend migration rate ζ_c_ using the HIPS model are later compared. When the differences between measured and calculated bend migration rates are less than the threshold value of 0.1 m/year, the corresponding *E* value is accepted as the calibrated riverbank erosion coefficient. Reach-averaged values of flow velocity, bankfull depth, and bankfull half channel width are calculated by fitting polynomial regressions to the measured local values (*37*, *50*).

## Supplementary Materials

### This PDF file includes:

Supplementary Text Figs. S1 to S3 References
