# Supplementary material for: High variability in flood discharge and stage accelerates river mobility
Source: Sci Adv. 2025 Nov 28;11(48):eadv7637. doi: 10.1126/sciadv.adv7637 (PMC12662209; doi:10.1126/sciadv.adv7637)
Supplement: Supplementary file 1 — Supplementary Text Figs. S1 to S3 References [file sciadv.adv7637_sm.pdf]

Science Advances
AAAS

Supplementary Materials for

High variability in flood discharge and stage accelerates river mobility

Chenliang Wu *et al.*

Corresponding author: Chenliang Wu, cwu21@tulane.edu; Wonsuck Kim, delta@yonsei.ac.kr;
Kyle M. Straub, kmstraub@tulane.edu

Sci. Adv. **11**, eadv7637 (2025)
DOI: 10.1126/sciadv.adv7637

This PDF file includes:

Supplementary Text
Figs. S1 to S3
References

Supplementary Text

Global dataset of river lateral mobility and discharge variability

River lateral migration rates are compiled from published datasets (*33*, *34*). Daily discharge records used for calculating discharge variability are downloaded from the Global Runoff Data Centre (https://portal.grdc.bafg.de/applications/public.html?publicuser=PublicUser#dataDownload/Stations) and the United States Geological Survey (https://waterdata.usgs.gov/nwis/rt).

Channel morphology and substrate

Channel bend sinuosity of the lowermost Mississippi River peaks downstream of Baton Rouge near RK 300 and then progressively decreases downstream (Fig. S1). The upstream channel section (RK 140 – 480) generally shows higher curvature compared to the downstream section (RK 0 – 140). The alluvial-bedrock transition lies between RK 230 and RK 290 (*48*, *84*). The bedrock-alluvial transition is located near RK 40 (*48*).

Levees constructed through overbank flow process likely comprise a large portion of the banklines of the LMR, even though it has been reported that the LMR incises into the underlying compacted Pleistocene sediments and Holocene mud particularly near bend thalwegs (*47*). For the upstream reach of the LMR, the levee crest is well above the surrounding floodplain (Fig. 1C) while the levee crest is only about 1 to 2 meters above sea level for the lower 100 RK reach (Fig. 1C). Considering the levees for the downstream reach extend to develop subaqueous portions, it is evident that the channel aggraded to offset the effect of subsidence and eustatic sea level rise, and to maintain the levee crest above sea level when this reach of the river developed over the past 500 to 700 years (*53*). A recent study revealed the rate of relative sea level rise (RSLR) to be more than 30 mm/year near the downstream reach of the river mainly driven by compaction of Holocene mud and local eustatic sea level rise (*85*). The eustatic sea level rise in the early Holocene was ~10 mm/year and during the end of Holocene was less than 1 mm/year (*86*), this suggests that the rate of RSLR during the formation of the downstream reach of the LMR was at least 20 mm/year. A back-of-envelope calculation of the amount of levee aggradation as the product of time (500 years) and RSLR (20 mm/year) plus the levee crest elevation above mean sea level (1 meter) yields 11 meters. This is around 55 percent of the reach-averaged bankfull channel depth of the LMR. This is a conservative estimate as the RSLR does not include the compaction of sediments in the top ~5 meters of the levee, which are more prone to compaction compared to the underlying sediments (*85*, *87*, *88*).

Stage record

Stage records were accessed from the US Army Corps of Engineers site (https://rivergages.mvr.usace.army.mil/WaterControl/new/layout.cfm) for the following gauging stations: Knox Landing, Red River Landing, Bayou Sara, St. Francisville, Baton Rouge,

Donaldsonville, College Point, Reserve, Bonnet Carre, New Orleans, IHNC Lock, Algiers Lock, Alliance, Pointe a la Hache, Empire, Venice, and West Bay.

The natural bankfull stage corresponds to the roll-over point on the rating curve (Fig. S2). As discharge increases above the bankfull discharge, the slope of the linear relationship between stage and discharge decreases (*6*). This suggests a natural bankfull stage of 30 ft at Baton Rouge.

Geotechnical boring

This study employs 1,656 geotechnical borings to investigate bank materials of the lowermost Mississippi River. These boring data were provided by the U.S. Army Corps of Engineers (USACE) and include sediment logs that can be used to evaluate grain size (*89*, *90*). Top elevations of these borings vary from -26 m to 28 m (NAVD 88). Sediment columns were collected while drilling and then used to measure grain size and test Atterberg limits (i.e., plastic and liquid limits) in the laboratory. Based on the grain size and Atterberg limits, sediments are classified following the Unified Soil Classification System (USCS) (*91*). The USCS classifies the sediments into three major divisions: coarse-grained sediments (gravels and sands) and fine-grained sediments (silts and clays), and highly organic soils (usually referred to as peat). These sediments are further divided into 15 sediment classes. Sediments at the geotechnical boring sites are mainly poorly-graded sands, silty sands, low plasticity silts, high plasticity clays, and low plasticity clays. Clay is the most abundant sediment type, comprising 53.8% of the total. The amounts of sand (25.8%) and silt (20.1%) are similar. No more than 0.3% of sediments are gravels or organic soils. Since silts from the borings mainly show low plasticity, they are grouped with sands as non-cohesive material (*92*).

The boring depth ranges 3 – 120 m with an average of 24 m. To document the sediment composition of the entire bank that is in contact with the river flow, sediment columns from borings are evaluated partially from the top to the depth of the local thalweg of the river (*37*, *50*). Percentages of different types of bank material (i.e., clay, silt and sand) of each boring are then calculated as the ratio between the total thickness of each material type to the thickness of the sediment column, within the depth range defined by the local thalweg (Fig. S3) (*37*).

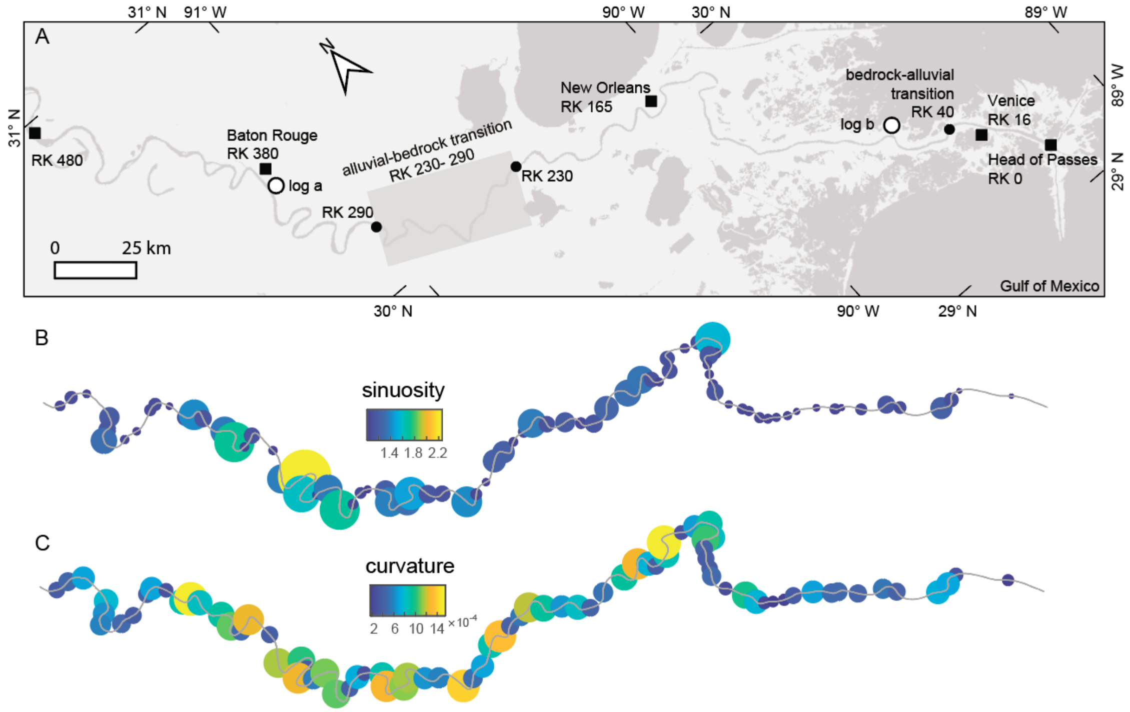

Figure S1. Planform morphology of the lowermost Mississippi River. (A) Map of the lowermost Mississippi River showing the locations of logs in Fig. 6A, alluvial-bedrock (marked by a gray box and black dots at the upstream and downstream ends) and bedrock-alluvial transitions (marked by a black dot). (B) Channel bend sinuosity. (C) Maximum bend curvature. Sinuosity and curvature are color-coded, and the circle size scales with the value of each metric.

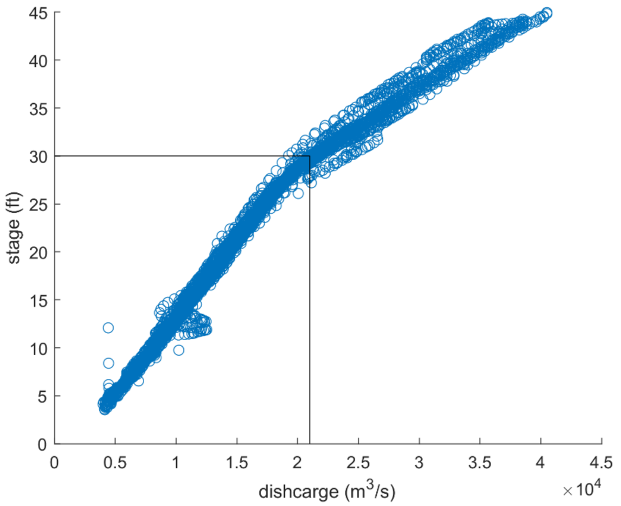

Figure S2. Rating curve at the Baton Rouge gauging station. The roll-over point on the plot corresponds to bankfull stage (horizontal line) and bankfull discharge (vertical line).

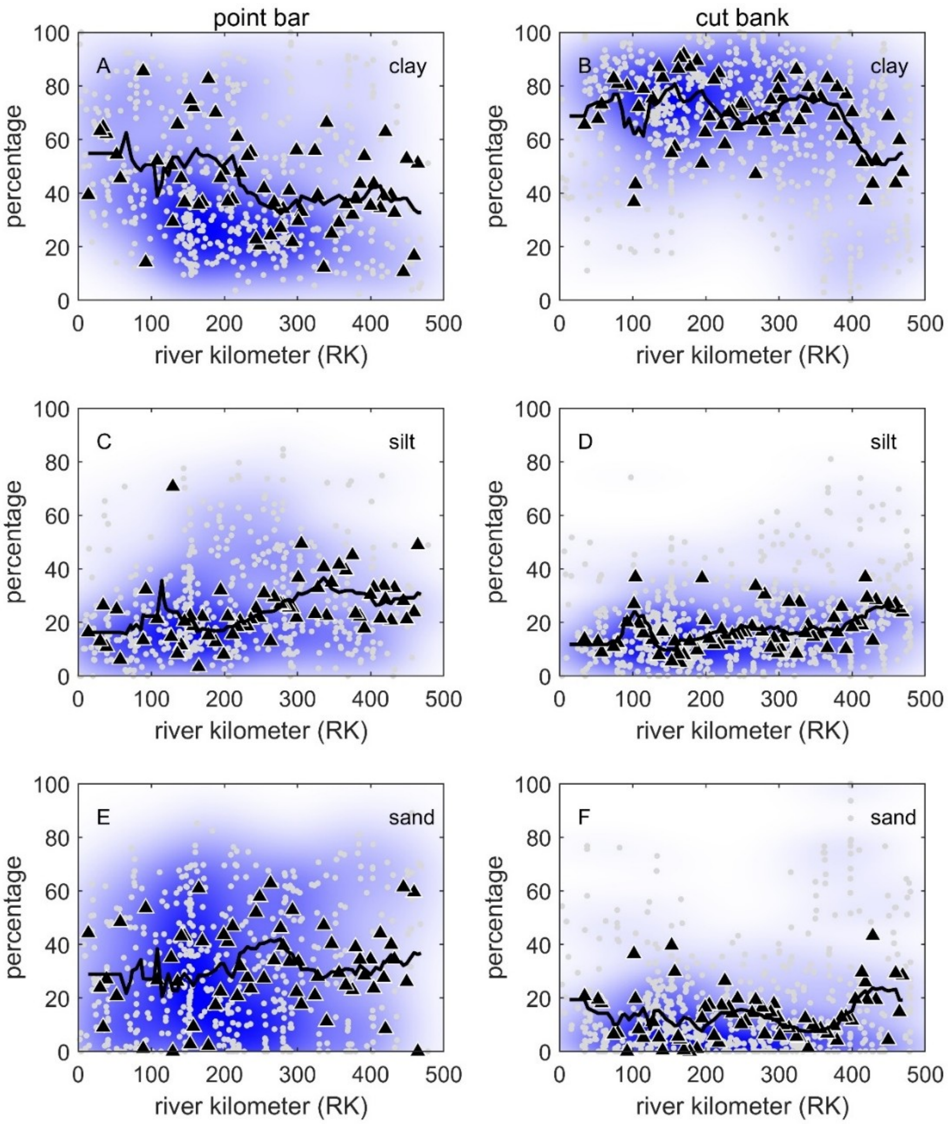

Figure S3. Spatial variabilities in innerbank (point bar) and outerbank (cut bank) material (i.e., clay, silt and sand) content for the lowermost Mississippi River. Spatial trends in clay, silt and sand for point bars (A, C and E) and cut banks (B, D, and F) are plotted against river kilometer (RK). Gray dots represent measurements from individual geotechnical boring. Triangles show bend-averaged values; black lines indicate the moving average. Heat maps depict the bivariate kernel density estimate.

REFERENCES AND NOTES

1. J. Best, Anthropogenic stresses on the world's big rivers. *Nat. Geosci.* **12**, 7–21 (2019).

2. K. Naito, G. Parker, Can bankfull discharge and bankfull channel characteristics of an alluvial meandering River be cospecified from a flow duration curve? *J. Geophys. Res. Earth* **124**, 2381–2401 (2019).

3. T. Y. Dong, J. A. Nittrouer, E. Il'icheva, M. Pavlov, B. McElroy, M. J. Czapiga, H. Ma, G. Parker, Controls on gravel termination in seven distributary channels of the Selenga River Delta, Baikal Rift basin, Russia. *Geol. Soc. Am. Bull.* **128**, 1297–1312 (2016).

4. L. B. Leopold, M. G. Wolman, J. P. Miller, *Fluvial Processes in Geomorphology* (Dover Publications Inc., 1964; https://pubs.usgs.gov/publication/70185663).

5. T. Dunne, L. B. Leopold, *Water in Environmental Planning* (Macmillan, 1978).

6. E. M. Lindroth, B. L. Rhoads, C. R. Castillo, J. A. Czuba, İ. Güneralp, D. Edmonds, Spatial variability in bankfull stage and riverbank elevations of lowland meandering rivers: Relation to rating curves and channel planform characteristics. *Water Resour. Res.* **56**, e2020WR027477 (2020).

7. IPCC, "Climate Change 2023: Synthesis Report. Contribution of Working Groups I, II and III to the Sixth Assessment Report of the Intergovernmental Panel on Climate Change [Core Writing Team, H. Lee and J. Romero (Eds.)]" (Geneva, Switzerland, 2023); https://doi.org/10.59327/IPCC/AR6-9789291691647.

8. J. Horacio, A. Ollero, I. Noguera, V. Fernández-Pasquier, Flooding, channel dynamics and transverse infrastructure: A challenge for Middle Ebro river management. *J. Maps* **15**, 310–319 (2019).

9. E. Wohl, S. N. Lane, A. C. Wilcox, The science and practice of river restoration. *Water Resour. Res.* **51**, 5974–5997 (2015).

10. S. E. Greco, A. K. Fremier, E. W. Larsen, R. E. Plant, A tool for tracking floodplain age land surface patterns on a large meandering river with applications for ecological planning and restoration design. *Landsc. Urban Plann.* **81**, 354–373 (2007).

11. K. Naito, G. Parker, Adjustment of self-formed bankfull channel geometry of meandering rivers: Modelling study. *Earth Surf. Process. Landf.* **45**, 3313–3322 (2020).

12. E. Eke, G. Parker, Y. Shimizu, Numerical modeling of erosional and depositional riverbankprocesses in migrating river bends with self-formed width: Morphodynamics of bar push and riverbankpull. *J. Geophys. Res. Earth* **119**, 1455–1483 (2014).

13. K. Asahi, Y. Shimizu, J. Nelson, G. Parker, Numerical simulation of river meandering with self-evolving banks. *Case Rep. Med.* **118**, 2208–2229 (2013).

14. J. M. Swartz, T. A. Goudge, D. C. Mohrig, Quantifying coastal fluvial morphodynamics over the last 100 years on the Lower Rio Grande, USA and Mexico. *J. Geophys. Res. Earth* **125**, e2019JF005443 (2020).

15. E. A. Barefoot, J. A. Nittrouer, K. M. Straub, Non-monotonic floodplain responses to changes in flooding intensity. *J. Geophys. Res. Earth* **126**, e2021JF006310 (2021).

16. C. R. Esposito, D. Di Leonardo, M. Harlan, K. M. Straub, Sediment storage partitioning in alluvial stratigraphy: The influence of discharge Variability. *J. Sediment. Res.* **88**, 717–726 (2018).

17. E. A. Barefoot, J. A. Nittrouer, B. Z. Foreman, E. A. Hajek, G. R. Dickens, T. Baisden, L. Toms, Evidence for enhanced fluvial channel mobility and fine sediment export due to precipitation seasonality during the Paleocene-Eocene thermal maximum. *Geology* **50**, 116–120 (2022).

18. A. Leenman, E. Greenberg, S. Moulds, M. Wortmann, L. Slater, V. Ganti, Accelerated river mobility linked to water discharge variability. *Geophys. Res. Lett.* **52**, e2024GL112899 (2025).

19. E. Greenberg, A. J. Chadwick, V. Ganti, A generalized area-based framework to quantify river mobility from remotely sensed imagery. *J. Geophys. Res. Earth* **128**, e2023JF007189 (2023).

20. M. R. Hansford, P. Plink-Björklund, E. R. Jones, Global quantitative analyses of river discharge variability and hydrograph shape with respect to climate types. *Earth Sci. Rev.* **200**, 102977 (2020).

21. C. R. Fielding, J. Alexander, J. P. Allen, The role of discharge variability in the formation and preservation of alluvial sediment bodies. *Sediment. Geol.* **365**, 1–20 (2018).

22. J. A. Nittrouer, D. Mohrig, M. Allison, Punctuated sand transport in the lowermost Mississippi River. *J. Geophys. Res. Earth* **116**, doi.org/10.1029/2011JF002026 (2011).

23. J. Han, W. Kim, Linking levee-building processes with channel avulsion: Geomorphic analysis for assessing avulsion frequency and channel reoccupation. *Earth Surf. Dynam.* **10**, 743–759 (2022).

24. K. B. J. Dunne, D. J. Jerolmack, What sets river width? *Sci. Adv.* **6**, eabc1505 (2020).

25. C. R. Constantine, T. Dunne, G. J. Hanson, Examining the physical meaning of the riverbankerosion coefficient used in meander migration modeling. *Geomorphology* **106**, 242–252 (2009).

26. J. E. Pizzuto, Bank erodibility of shallow sandbed streams. *Earth Surf. Process. Landf.* **9**, 113–124 (1984).

27. K. M. Konsoer, B. L. Rhoads, E. J. Langendoen, J. L. Best, M. E. Ursic, J. D. Abad, M. H. Garcia, Spatial variability in riverbankresistance to erosion on a large meandering, mixed bedrock-alluvial river. *Geomorphology* **252**, 80–97 (2016).

28. C. Parker, A. Simon, C. R. Thorne, The effects of variability in riverbankmaterial properties on riverbank stability: Goodwin Creek, Mississippi. *Geomorphology* **101**, 533–543 (2008).

29. J. P. Julian, R. Torres, Hydraulic erosion of cohesive riverbanks. *Geomorphology* **76**, 193–206 (2006).

30. K. B. J. Dunne, P. E. Arratia, D. J. Jerolmack, A new method for in situ measurement of the erosion threshold of river channels. *Water Resour. Res.* **58**, e2022WR032407 (2022).

31. W. M. van Dijk, W. I. van de Lageweg, M. G. Kleinhans, Formation of a cohesive floodplain in a dynamic experimental meandering river. *Earth Surf. Process. Landf.* **38**, 1550–1565 (2013).

32. J. Peakall, P. J. Ashworth, J. L. Best, Meander-bend evolution, alluvial architecture, and the role of cohesion in sinuous river channels: A flume study. *J. Sediment. Res.* **77**, 197–212 (2007).

33. C. Wu, W. Kim, R. Herring, B. T. Cardenas, T. Y. Dong, H. Ma, A. Moodie, J. A. Nittrouer, F. Tsai, A. Li, Lowland river sinuosity on Earth and Mars set by the pace of meandering and avulsion. *Nat. Geosci.* **16**, 747–753 (2023).

34. E. Greenberg, V. Ganti, The pace of global river meandering influenced by fluvial sediment supply. *Earth Planet. Sci. Lett.* **634**, 118674 (2024).

35. Z. Sylvester, P. Durkin, J. A. Covault, High curvatures drive river meandering. *Geology* **47**, 263–266 (2019).

36. H. E. Beck, N. E. Zimmermann, T. R. McVicar, N. Vergopolan, A. Berg, E. F. Wood, Present and future Köppen-Geiger climate classification maps at 1-km resolution. *Sci Data* **5**, 180214 (2018).

37. J. A. Nittrouer, J. Shaw, M. P. Lamb, D. Mohrig, Spatial and temporal trends for water-flow velocity and bed-material sediment transport in the lower Mississippi River. *Geol. Soc. Am. Bull.* **124**, 400–414 (2012).

38. P. F. Hudson, R. H. Kesel, Channel migration and meander-bend curvature in the lower Mississippi River prior to major human modification. *Geology* **28**, 531–534 (2000).

39. V. Smith, J. Mason, D. Mohrig, Reach-scale changes in channel geometry and dynamics due to the coastal backwater effect: The lower Trinity River, Texas. *Earth Surf. Process. Landf.* **45**, 565–573 (2020).

40. H. J. Hassenruck-Gudipati, P. Passalacqua, D. Mohrig, Natural levees increase in prevalence in the backwater zone: Coastal Trinity River, Texas, USA. *Geology* **50**, 1068–1072 (2022).

41. M. P. Lamb, J. A. Nittrouer, D. Mohrig, J. Shaw, Backwater and river plume controls on scour upstream of river mouths: Implications for fluvio-deltaic morphodynamics. *J. Geophys. Res.* **117**, doi.org/10.1029/2011JF002079 (2012).

42. Z. Shen, T. E. Törnqvist, B. Mauz, E. L. Chamberlain, A. G. Nijhuis, L. Sandoval, Episodic overbank deposition as a dominant mechanism of floodplain and delta-plain aggradation. *Geology* **43**, 875–878 (2015).

43. K. Zhao, G. Coco, Z. Gong, S. E. Darby, S. Lanzoni, F. Xu, K. Zhang, I. Townend, A. Review, A review on bank retreat: Mechanisms, observations, and modeling. *Rev. Geophys.* **60**, e2021RG000761 (2022).

44. J. A. Constantine, T. Dunne, J. Ahmed, C. Legleiter, E. D. Lazarus, Sediment supply as a driver of river meandering and floodplain evolution in the Amazon Basin. *Nat. Geosci.* **7**, 899–903 (2014).

45. A. Ielpi, M. G. A. Lapôtre, A tenfold slowdown in river meander migration driven by plant life. *Nat. Geosci.* **13**, 82–86 (2020).

46. A. Finotello, A. Ielpi, M. G. A. Lapôtre, E. D. Lazarus, M. Ghinassi, L. Carniello, S. Favaro, D. Tognin, A. D'Alpaos, Vegetation enhances curvature-driven dynamics in meandering rivers. *Nat. Commun.* **15**, 1968 (2024).

47. J. A. Nittrouer, D. Mohrig, M. A. Allison, A.-P. B. Peyret, The lowermost Mississippi River: A mixed bedrock-alluvial channel. *Sedimentology* **58**, 1914–1934 (2011).

48. E. Viparelli, J. A. Nittrouer, G. Parker, Modeling flow and sediment transport dynamics in the lowermost Mississippi River, Louisiana, USA, with an upstream alluvial-bedrock transition

and a downstream bedrock-alluvial transition: Implications for land building using engineered diversions. *J. Geophys. Res. Earth* **120**, 534–563 (2015).

49. H. Rouse, Modern conceptions of the mechanics of fluid turbulence. *Trans. Am. Soc. Civil Eng.* **102**, 463–505 (1937).

50. O. P. Harmar, "Morphological and process dynamics of the Lower Mississippi River," thesis, University of Nottingham (2004).

51. H. Ma, J. A. Nittrouer, K. Naito, X. Fu, Y. Zhang, A. J. Moodie, Y. Wang, B. Wu, G. Parker, The exceptional sediment load of fine-grained dispersal systems: Example of the Yellow River, China. *Sci. Adv.* **3**, e1603114 (2017).

52. R. Osborn, K. B. J. Dunne, T. Ashley, J. A. Nittrouer, K. Strom, The flocculation state of mud in the lowermost freshwater reaches of the Mississippi River: Spatial distribution of sizes, seasonal changes, and their impact on vertical concentration profiles. *J. Geophys. Res. Earth* **128**, e2022JF006975 (2023).

53. J. W. Day, D. F. Boesch, E. J. Clairain, G. P. Kemp, S. B. Laska, W. J. Mitsch, K. Orth, H. Mashriqui, D. J. Reed, L. Shabman, C. A. Simenstad, B. J. Streever, R. R. Twilley, C. C. Watson, J. T. Wells, D. F. Whigham, Restoration of the Mississippi Delta: Lessons from hurricanes Katrina and Rita. *Science* **315**, 1679–1684 (2007).

54. D. A. Edmonds, S. C. Toby, C. G. Siverd, R. Twilley, S. J. Bentley, S. Hagen, K. Xu, Land loss due to human-altered sediment budget in the Mississippi River Delta. *Nat. Sustain.* **6**, 644–651 (2023).

55. S. Patsinghasanee, I. Kimura, Y. Shimizu, M. Nabi, Experiments and modelling of cantilever failures for cohesive riverbanks. *J. Hydraul. Res.* **56**, 76–95 (2016).

56. G. Parker, Y. Shimizu, G. V. Wilkerson, E. C. Eke, J. D. Abad, J. W. Lauer, C. Paola, W. E. Dietrich, V. R. Voller, A new framework for modeling the migration of meandering rivers. *Earth Surf. Process. Landf.* **36**, 70–86 (2011).

57. M. M. Douglas, K. B. J. Dunne, M. P. Lamb, Sediment entrainment and slump blocks limit permafrost riverbank erosion. *Geophys. Res. Lett.* **50**, e2023GL102974 (2023).

58. M. G. Donat, A. L. Lowry, L. V. Alexander, P. A. O'Gorman, N. Maher, More extreme precipitation in the world's dry and wet regions. *Nat. Clim. Chang.* **6**, 508–513 (2016).

59. L. M. Bouwer, J. E. Vermaat, J. C. J. H. Aerts, Regional sensitivities of mean and peak river discharge to climate variability in Europe. *J. Geophys. Res. Atmos.* **113**, (2008).

60. N. W. Arnell, S. N. Gosling, The impacts of climate change on river flow regimes at the global scale. *J. Hydrol.* **486**, 351–364 (2013).

61. N. Wanders, Y. Wada, H. a. J. Van Lanen, Global hydrological droughts in the 21st century under a changing hydrological regime. *Earth Syst. Dyn.* **6**, 1–15 (2015).

62. T. Y. Dong, T. A. Goudge, Quantitative relationships between river and channel-belt planform patterns. *Geology* **50**, 1053–1057 (2022).

63. C. J. Feeney, R. C. Chiverrell, H. G. Smith, J. M. Hooke, J. R. Cooper, Modelling the decadal dynamics of reach-scale river channel evolution and floodplain turnover in CAESAR-Lisflood. *Earth Surf. Process. Landf.* **45**, 1273–1291 (2020).

64. J. M. Hooke, Magnitude and distribution of rates of river riverbankerosion. *Earth Surf. Process.* **5**, 143–157 (1980).

65. E. C. Geyman, Y. Ke, J. S. Magyar, J. N. Reahl, V. Soldano, N. D. Brown, A. J. West, W. W. Fischer, M. P. Lamb, Scaling laws for sediment storage and turnover in river floodplains. *Sci. Adv.* **11**, DOI: 10.1126/sciadv.adu8574 (2025).

66. M. A. Torres, A. B. Limaye, V. Ganti, M. P. Lamb, A. J. West, W. W. Fischer, Model predictions of long-lived storage of organic carbon in river deposits. *Earth Surf. Dyn.* **5**, 711–730 (2017).

67. M. E. Huffman, J. E. Pizzuto, S. M. Trampush, J. A. Moody, D. M. Schook, H. J. Gray, S. A. Mahan, Floodplain sediment storage timescales of the laterally confined meandering Powder River, USA. *J. Geophys. Res. Earth* **127**, e2021JF006313 (2022).

68. P. L. Heller, D. Ratigan, S. Trampush, A. Noda, B. McElroy, J. Drever, S. Huzurbazar, Origins of bimodal stratigraphy in fluvial deposits: An example from the Morrison Formation (Upper Jurassic), Western U.S.A. *J. Sediment. Res.* **85**, 1466–1477 (2015).

69. E. P. Chamberlin, E. A. Hajek, Fine-sediment supply can control fluvial deposit architecture: An example from the Blackhawk Formation-Castlegate Sandstone Transition, Upper Cretaceous, Utah, USA. *Depos. Record* **20**, doi.org/10.2110/001c.36334 (2022).

70. J. Zhang, P. M. Burgess, D. Granjeon, R. Steel, Can sediment supply variations create sequences? Insights from stratigraphic forward modelling. *Basin Res.* **31**, 274–289 (2019).

71. H. Wu, Y. Ji, C. Wu, G. Duclaux, H. Wu, C. Gao, L. Li, L. Chang, Stratigraphic response to spatiotemporally varying tectonic forcing in rifted continental basin: Insight from a coupled tectonic-stratigraphic numerical model. *Basin Res.* **31**, 311–336 (2019).

72. C. M. Hughes, B. T. Cardenas, T. A. Goudge, D. Mohrig, Deltaic deposits indicative of a paleo-coastline at Aeolis Dorsa, Mars. *Icarus* **317**, 442–453 (2019).

73. A. M. Fernandes, T. E. Törnqvist, K. M. Straub, D. Mohrig, Connecting the backwater hydraulics of coastal rivers to fluvio-deltaic sedimentology and stratigraphy. *Geology* **44**, 979–982 (2016).

74. C. Wu, M. Kim, W. Kim, Natural levees increase in prevalence in the backwater zone: Coastal Trinity River, Texas, USA: COMMENT. *Geology* **51**, e564 (2023).

75. C. M. Hughes, J. B. Shaw, A. M. Fernandes, T. E. Swanson, Stratigraphic evidence of backwater morphodynamics and lowland river deltas in the Northern Hemisphere of Mars. *Geophys. Res. Lett.* **52**, e2024GL112957 (2025).

76. G. Stucky de Quay, T. A. Goudge, E. S. Kite, C. I. Fassett, S. D. Guzewich, Limits on runoff episode duration for early Mars: Integrating lake hydrology and climate models. *Geophys. Res. Lett.* **48**, e2021GL093523 (2021).

77. P. L. Wiberg, D. M. Rubin, Bed roughness produced by saltating sediment. *J. Geophys. Res. Oceans* **94**, 5011–5016 (1989).

78. J. D. Smith, S. R. McLean, Spatially averaged flow over a wavy surface. *J. Geophys. Res.* **82**, 1735–1746 (1977).

79. W. E. Dietrich, Settling velocity of natural particles. *Water Resour. Res.* **18**, 1615–1626 (1982).

80. A. J. Moodie, J. A. Nittrouer, H. Ma, B. N. Carlson, Y. Wang, M. P. Lamb, G. Parker, Suspended sediment-induced stratification inferred from concentration and velocity profile measurements in the Lower Yellow River, China. *Water Resour. Res.* **58**, e2020WR027192 (2022).

81. K. Hasegawa, "Computer simulation of the gradual migration of meandering channels" in *Proceedings of the Hokkaido Branch, Japan Society of Civil Engineering* (Japan Society of Civil Engineering, Tokyo, Japan, 1977), pp. 197–202.

82. S. Ikeda, G. Parker, K. Sawai, Bend theory of river meanders. Part 1. Linear development. *J. Fluid Mech.* **112**, 363–377 (1981).

83. T. Sun, P. Meakin, T. Jøssang, K. Schwarz, A simulation model for meandering rivers. *Water Resour. Res.* **32**, 2937–2954 (1996).

84. J. A. Nittrouer, Backwater hydrodynamics and sediment transport in the lowermost Mississippi River Delta: Implications for the development of fluvial-deltaic landforms in a large lowland river. *IAHS-AISH Publication* **358**, 48–61 (2013).

85. G. Li, T. E. Törnqvist, S. Dangendorf, Real-world time-travel experiment shows ecosystem collapse due to anthropogenic climate change. *Nat. Commun.* **15**, 1226 (2024).

86. J. B. Anderson, D. J. Wallace, A. B. Rodriguez, A. R. Simms, K. T. Milliken, "Holocene evolution of the Western Louisiana–Texas Coast, USA: Response to sea-level rise and climate change" in *Geological Society of America Memoir 221* (Geological Society of America, 2022; https://pubs.geoscienceworld.org/gsa/books/book/2361/chapter/133567225/Holocene-Evolution-of-the-Western-Louisiana-Texas), pp. 1–81.

87. K. L. Jankowski, T. E. Törnqvist, A. M. Fernandes, Vulnerability of Louisiana's coastal wetlands to present-day rates of relative sea-level rise. *Nat. Commun.* **8**, 14792 (2017).

88. E. L. Chamberlain, Z. Shen, W. Kim, S. McKinley, S. Anderson, T. E. Törnqvist, Does load-induced shallow subsidence inhibit delta growth? *J. Geophys. Res. Earth* **126**, e2021JF006153 (2021).

89. A. Li, F. T.-C. Tsai, B. T. Yuill, C. Wu, A three-dimensional stratigraphic model of the Mississippi River Delta, USA: Implications for river deltaic hydrogeology. *Hydrgeol. J.* **28**, 2341–2358 (2020).

90. Y. Song, F. T.-C. Tsai, B. J. Minsley, C. Wu, E. Heggy, Quantitative subsurface characterization illuminates the origin of the Mississippi River Valley alluvial aquifer. *Commun. Earth Environ.* **6**, 646 (2025).

91. ASTM, Standard Practice for Classification of Soils for Engineering Purposes (Unified Soil Classification System) (2018). https://doi.org/10.1520/D2487-17.

92. S. Wang, R. Luna, J. Yang, Effect of plasticity on shear behavior of low-plasticity fine-grained soil. *J. Mater. Civ. Eng.* **29**, 04016228 (2017).
